# Food for Reflection

**Published:** 1887-07

**Authors:** 


### Food For Reflection.

AN ESSAY UPON HOW WE INVITE AN UNWELCOME VISITOR.

There is an old gentleman roaming around through the pleasant valleys of the earth. His garments are specially adapted to hot weather, and he always travels on foot. He carries in his hand a scythe, and his name is Death. He has no first name, nor middle name, nor does he add L.L.D. to his signature, but just registers as Death and no questions are asked. He bivouacs sometimes on the grassy plain and sometimes in the gilded parlor; and wherever his campfire burns there will be a funeral. While other travelers occupy reclining chair cars, or covered buggies, the old man trots along on foot, and neither rivers or barbed wire fences will stop him. When he marks a man he just comes up behind him, winds his scythe about him, and then the the undertaker is sent for, and Death goes across the street to gather in the boy who has been eating green apples. His coming sends a cold chill over us; his shadow falls upon our hearts like a hand of ice, and his cold breath covers our foreheads with dampness; as we lie on the shore of the wide damp river, and listen to the faint roar of the receding world, his soft but relentless arm is about us, and he bears us gently away, from all the woes, the shadows, the trials of a world we have come to hate. He never calls twice for the same man. His coming is dreaded, yet people invite him. His name pales the bright cheek of health, and quivers the tired hand of age, still he is invited. The young woman who sits down and eats ice cream for half an hour at a time and mixes it with lemonade and finishes off with peanuts is writing an invitation to Death. When she paints her young cheeks with poisonous drugs and lubricates her tresses with hair restoratives, she is beckoning to Death. When she compresses her body in tight-drawn dresses, until every breath she draws sends a pang through her silly bosom; when she swallows poison with a gaudy label, to improve her complexion; when she stands out in the moonlight in her gauzy dress, with the dew settling upon her; when she dances through the hours which nature demands for sleep, and sleeps through the hours which nature demands for exercise, she is sealing the missive that calls Death to her bedside, and you may rest assured, Arabella, that he will come. Death never makes apologies when you send him your invitation, but he sharpens his scythe on the nearest grindstone and strikes for your house by the nearest road. Look at the young dude with a frame like an extenuated string; with a breast like a spring chicken, and legs like a stork; if you talk about dissolution or similar cheerful subjects to him, he offers you a cigar to stop, and there isn't money enough in Atchison to coax him to climb over a fence into a graveyard after dark. He is, nevertheless, soliciting a visit from Death; he smokes cigarettes which poison by inches; he drinks soda water, which converts blood into water; he eats candies, which are made of terra alba, baryta, and prussic acid, and every day that passes he writes another line in the letter which will shortly be sent to Death, and which will be answered in person. The small boy dreads the scythe; yet he loads his interior economy with unripe fruit, and smokes cigars extracted from the gutter, and devours whole pies at one sitting. Then, when the soft shades of evening have fallen, and the plaintive frog is wailing a fitting requiem beneath his window, that boy will be found writhing in his bed. And when its agony is at its worst, a stranger will enter the room; no doors open to announce his entrance; the subdued lamplight does not betray his presence; no mortal eye can see him, but there, in the solemn hush of the summer eve, he raises his scythe on high, and in a few minutes there is a dead boy lying there, while the old man slides down the eave trough and goes over to talk with the servant girl who is trying to light the fire with kerosene. Over in Spain he has been putting in extra time, and the dull thud of his mowing machine is heard on every hand. It would be seasonable to give him the cold shoulder by abating all nuisances in town, cleaning up the alleys, and filling our hats with ice, for should he ever get started to work here, the hospitals will be filled with victims, and and an extra force of sextons will have to be engaged.—*Atchison* (*Kansas*) *Globe.*

THE *Century Magazine* believes that "we waste enough in ignorant buying and bad cooking to sustain another nation equally numerous." We also waste enough medicine, by taking it when not needed, to properly treat the diseases of two equally numerous nations.

"YOU take a basin of water, place your finger in it for twenty or thirty seconds, take it out and look at the hole that is left. The size of that hole represents about the impression that advice makes on a young man's mind.

"Don't depend too much on your family—the dead part I mean. The world wants live men; it has no use for dead ones. Queen Victoria can trace her ancesters back in a direct line to William the Conqueror. If you cannot get further back than your father you are better off. Your father was a better man in his time than old William. He had better clothes to wear, better food to eat, and was better housed.

"If you are a diamond be sure that you will be found. Cheek, brass, or gall never gets ahead of merit.

"I love a young man who is straight-forward. Ask for what you want. If you want to marry a rich man's daughter or borrow $500 from him, ask him for it; it amounts to the same thing in the end. It is always better to astonish a man than to bore him.

"Remember that in the morning of life come the hard-working days. Hard work never killed a man. It's fun, recreation, relaxation, holiday, that kill. The fun that results in a head the next morning so big that a tub could hardly cover it, is what kills. Hard work never does.

"Those who come after us have to work just as hard as we do. When I shovel the snow off my side walk, if perchance I take a three-quarter piece off my neighbor's walk, I put it back, because if I didn't I should be doing him an injustice.

"You can't afford to do anything but what is good. You are on dress parade all the time.

"Don't be afraid of pounding persistently at one thing. Don't be afraid of being called a one-idea man or a crank. If you have one idea, you have one more than most men have. It takes a smart man to be a crank.

